# Improving Uncertainty Estimation With Semi-Supervised Deep Learning for COVID-19 Detection Using Chest X-Ray Images

**DOI:** 10.1109/ACCESS.2021.3085418

**Published:** 2021-06-02

**Authors:** Saul Calderon-Ramirez, Shengxiang Yang, Armaghan Moemeni, Simon Colreavy-Donnelly, David A. Elizondo, Luis Oala, Jorge Rodríguez-Capitán, Manuel Jiménez-Navarro, Ezequiel López-Rubio, Miguel A. Molina-Cabello

**Affiliations:** School of Computer Science and InformaticsDe Montfort University 4487 Leicester LE1 9BH U.K.; Instituto Tecnologico de Costa Rica 27942 Cartago 30101 Costa Rica; School of Computer ScienceUniversity of Nottingham 6123 Nottingham NG8 1BB U.K.; XAI GroupArtificial Intelligence DepartmentFraunhofer Heinrich Hertz Institute 10587 Berlin Germany; CIBERCVHospital Universitario Virgen de la Victoria 16867 29010 Málaga Spain; Department of Computer Languages and Computer ScienceUniversity of Málaga 29071 Málaga Spain; Instituto de Investigación Biomédica de Mñlaga (IBIMA) 29010 Málaga Spain

**Keywords:** Uncertainty estimation, Coronavirus, Covid-19, chest x-ray, computer aided diagnosis, semi-supervised deep learning, MixMatch

## Abstract

In this work we implement a COVID-19 infection detection system based on chest X-ray images with uncertainty estimation. Uncertainty estimation is vital for safe usage of computer aided diagnosis tools in medical applications. Model estimations with high uncertainty should be carefully analyzed by a trained radiologist. We aim to improve uncertainty estimations using unlabelled data through the MixMatch semi-supervised framework. We test popular uncertainty estimation approaches, comprising Softmax scores, Monte-Carlo dropout and deterministic uncertainty quantification. To compare the reliability of the uncertainty estimates, we propose the usage of the Jensen-Shannon distance between the uncertainty distributions of correct and incorrect estimations. This metric is statistically relevant, unlike most previously used metrics, which often ignore the distribution of the uncertainty estimations. Our test results show a significant improvement in uncertainty estimates when using unlabelled data. The best results are obtained with the use of the Monte Carlo dropout method.

## I. Introduction

The COVID-19 pandemic is putting significant pressure on governmental health systems, as the number of cases grows exponentially [1]. Furthermore, the availability of medical staff is lowered as they also get infected by the virus, reducing the overall capacity of hospitals and clinics [1]. The accurate and widespread detection of infected subjects is of great importance to control the growth of the disease [2]. The usage of medical imaging can be an alternative tool when other methods like Real-time Reverse Transcription Polymerase Chain Reaction (RT-PCR) testing become more expensive as less resources are available to supply the growing demand [3]. The usage of computed tomography and X-ray based tests for COVID-19 detection has been studied in [4]–[6], reporting mixed sensitivity and accuracy in the case of X-ray imaging based solutions. However, the usage of X-ray images is ubiquitous, as this technology is usually cheaper and more widely available [7].

X-ray chest imaging is in general more widely accessible when compared to computed tomography imaging [7]. Furthermore, the low availability of medical staff to sample and analyze the medical images can increase the costs of this alternative solution, especially in low resource environments [8]. For example, in India, with a population of around 1.44 billion, approximately one radiologist for every 100,000 people is currently available [8]. This increases the need of X-ray based COVID-19 computer aided diagnosis tools.

The application of deep learning based models to estimate the prevalence of COVID-19 from X-ray images has recently been explored, with different deep learning architectures reporting high test accuracy [9], [10]. Given the lack of high quality labeled data, semi-supervised methods have also been implemented to perform COVID-19 detection, making use of cheaper unlabelled data to improve the model’s accuracy [11], [12].

Along with high model accuracy, Artificial Intelligence (AI) based solutions should also provide explainable decisions to increase reliability, especially in the medical domain [13], [14]. Model uncertainty estimation is a common approach to increase model interpretability and safety in use [13], [15]. The estimation of model uncertainty allows the user to interpret how sure or confident is the model for a specific prediction. In the context of COVID-19 detection using X-ray images, an estimation with high uncertainty should justify further tests to be done in the subject. This enforces safety upon the usage of a computer aided diagnosis system, as low-confidence predictions are quantitatively estimated by the system itself.

In this work we focus in the measurement and improvement of uncertainty estimations for a deep learning model designed to identify COVID-19 infection using X-ray images. We aim to improve uncertainty estimations by using unlabelled data. Using unlabelled data is an useful approach when using datasets with a low number of high quality labelled data. This is a frequent setting during the onset of a pandemic. Moreover, for a statistically significant comparison of the tested uncertainty estimation methods, we propose a novel density function based divergence approach.

## II. State of the Art

### A. Predictive Uncertainty Estimation

Predictive uncertainty estimation (or simply referred as uncertainty estimation in this work) for machine learning models has been widely studied in the literature [16]. In general, uncertainty sources can be categorized in aleatoric and epistemic. Aleatoric uncertainty refers to the uncertainty inherent in the measurements [17]. In conditional distribution terms, it refers to the distribution of the target variables with a given set of measured features. Aleatoric uncertainty cannot be reduced by taking a larger sample of features within the same distribution [17]. Epistemic uncertainty refers to the model’s parameters uncertainty caused by the limited sample size used to build the model (or lack of knowledge of the feature space) [17]. Therefore, epistemic uncertainty can be diminished by sampling a larger dataset, specially collecting data in the sparser regions [17]. In the context of Semi-supervised Deep Learning (SSDL), epistemic uncertainty can be considered to be more important, as labeled data are usually very scarce when SSDL is used. Unlabelled data might lower epistemic uncertainty, usually less effectively as target information is missing [17].

In this work we analyze simple and straightforward uncertainty estimation methods. The tested methods were selected based on their post-hoc capacity, i.e. their ability to leave the original deep learning architecture intact and not require any re-training of the model.

The Softmax function, typically used as an activation function in the output layer of a neural network, is among the basic methods for uncertainty estimation. Assume a multi-class discrimination problem in $i = 1, \ldots, C$ classes [18]. Take the array of model outputs $\boldsymbol {y}_{j}=f_{\boldsymbol {\theta }}(\boldsymbol {x}_{j})$ with network weights $\boldsymbol {\theta }$ for a given input $\boldsymbol {x}_{j}$. The Softmax function approximates a density function $\boldsymbol {p}$ as follows:

\begin{equation*} p_{i}=\frac {\exp \left ({y_{i,j}}\right)}{\sum _{k}\exp \left ({y_{k,j}}\right)} \tag{1}\end{equation*}

Therefore, the output of the Softmax function for a specific output unit $i$ can be interpreted as a proxy for model confidence for class $i$, given a specific input observation $\boldsymbol {x}_{j}$. Either the highest $p_{i}$ for the estimated class or the entropy over $\boldsymbol {p}$ can be used for uncertainty estimation. However, authors in [19] highlight how neural networks are typically overconfident in their predictions, leading to poor uncertainty estimations.

To address this, authors in [20] propose to post-process the Softmax’s confidence outputs, by implementing an additional temperature parameter $T$ in the Softmax function:

\begin{equation*} p_{i}=\frac {\exp \left ({y_{i,j}/T}\right)}{\sum _{k}\exp \left ({y_{k,j}/T}\right)}.\tag{2}\end{equation*}

To find the optimum $T$ leading to better uncertainty estimates, the authors propose to minimize the negative log likelihood, encouraging the model to assign high confidence to correct classes only (ignoring incorrect classes). This means that an additional optimization step is needed.

Authors in [19] propose an alternative approach to avoid the Softmax based uncertainty estimates, known as Monte Carlo Dropout (MCD). In their method forward passes through $M$ perturbed models $\boldsymbol {y}_{j,m}=f_{\boldsymbol {\theta '}_{m}}\left ({\boldsymbol {x}_{j} }\right)$ with perturbed weights $\boldsymbol {\theta '}_{m}$ are performed. This way, epistemic uncertainty is modeled with a distribution of the model’s weights [21]. The approach estimates the dispersion $\sigma _{\textrm {model}}\left ({\boldsymbol {x}_{j} }\right)$ of $M$ evaluations of the perturbed model, for the same input observation $\boldsymbol {x}_{j}$:

\begin{equation*} \sigma _{\textrm {model}}^{2}\left ({\boldsymbol {x}_{j}}\right)=\frac {1}{M}\sum _{m=1}^{M}\sum _{k=1}^{K}\left ({y_{m,j,k}-\bar {y}_{j,k}}\right)^{2}. \tag{3}\end{equation*}

The calculation of the dispersion or the distribution of the outputs can be summed for all the output units $k=1,\ldots,K$, or only the unit with the highest output can be taken into account.

Another recent method in [22] relying on the feature representations of the training data, was proposed for uncertainty estimation and Out of Distribution (OOD) data detection. This method is known as Deterministic Uncertainty Quantification (DUQ). For a set of feature centroids $E=\left \{{ \boldsymbol {e}_{1},\ldots,\boldsymbol {e}_{C}}\right \}$ calculated using the training data, uncertainty is calculated using the distance from each centroid to the input observation $\boldsymbol {x}_{j}$, with a radial basis kernel $K_{i}$:

\begin{equation*} K_{i}\left ({f_{\boldsymbol {\theta }}\left ({\boldsymbol {x}_{j}}\right),\boldsymbol {e}_{i}}\right)=\exp \left ({-\frac {\left \Vert{ W_{i}f_{\boldsymbol {\theta }}\left ({\boldsymbol {x}_{j}}\right)-\boldsymbol {e}_{i}}\right \Vert _{2}^{2}}{2\sigma ^{2}}}\right) \tag{4}\end{equation*}

where $W_{i}$ stands for a weight matrix, tuned to encourage feature insensitivity per class, thereby minimizing feature collapse [22]. The uncertainty is then estimated as the maximum class centroid distance in the feature space:

\begin{equation*} \underset {i}{\arg \max }~K_{i}\left ({f_{\boldsymbol {\theta }}\left ({\boldsymbol {x}_{j}}\right),\boldsymbol {e}_{i}}\right).\tag{5}\end{equation*}

The authors of the DUQ method claim that their approach measures both the epistemic and aleatoric uncertainty. Epistemic uncertainty is modeled through the construction of the feature centroids and the kernel $K_{i}$, which can improve as more data is available. The measurement of the centroids also includes aleatoric uncertainty [22].

Other popular uncertainty approaches include deep ensembles [23] and interval networks [24]. These methods require additional training steps, increasing complexity, and are often impractical when no access to the original training data set is possible.

### B. Uncertainty Estimation for Reliable Medical Imaging Analysis and COVID-19 Detection

Uncertainty estimation has been implemented in the literature to increase the reliability of medical imaging analysis systems. For example, in [25] uncertainty estimation is implemented for a diabetic retinopathy diagnosing system. A MCD based approach for uncertainty estimation was implemented. The system was evaluated using rejection plots, which calculate the average accuracy for the data rejected by using different uncertainty thresholds. Furthermore, the reliability was evaluated by measuring the impact of referring samples to further manual inspection during clinical usage.

In [26], a Bayesian deep learning approach was implemented to segment retinal optical coherence tomographies. The Bayesian model is able to estimate an uncertainty map, used to post-process the segmentation. Neither a comparison to other uncertainty methods nor the usage of uncertainty metrics was performed in the study.

As for COVID-19 detection, a system with uncertainty assessment was proposed in [27]. By providing practitioners with a confidence factor of the prediction, the overall reliability of the system is said to be improved. A high correlation between the prediction accuracy of the model and the level of uncertainty was reported in [27]. The data set used for positive COVID-19 cases uses the repository of [28], and normal X-ray readings were collected from [29].

Perhaps the most similar previous method to our proposed approach is the pre-published work of [30]. The authors write on the importance of measuring model uncertainty for COVID-19 detection from chest X-ray images. They compared three popular uncertainty estimation approaches, namely ensemble networks, Monte Carlo dropout and a combination of both approaches. An objective uncertainty estimation metric is also proposed, as the authors found a lack of metrics to compare uncertainty estimation methods. We agree on this gap in the literature, however we think that the metric should allow to compare not only different uncertainty estimation methods, but also several uncertainty estimations with different deep learning architectures, leading to different accuracy measurements, with statistical significance. Reference [30] proposed a confusion matrix approach which does not hold statistical meaning by itself. Therefore, in our work, we propose an alternative metric to compare different uncertainty estimation methods and assess the impact of semi-supervised learning on uncertainty estimation.

### C. Semi-Supervised Learning With MixMatch

In this work, we explore the recent and successful SSDL method referred to as MixMatch [31]. It creates a set of pseudo-labels, and also implements an unsupervised regularization term. The consistency loss implemented uses the pseudo-labels for the unlabelled dataset $X_{u}$ to train the model. To calculate the pseudo-labels, the average model output of a perturbed input $\boldsymbol {x}_{j}$ is used:

\begin{equation*} \widehat {\boldsymbol {y}}{}_{j}=\frac {1}{K}\sum _{\eta =1}^{K}f_{\boldsymbol {w}}\left ({\Psi ^{\eta }\left ({\boldsymbol {x}_{j}}\right)}\right).\tag{6}\end{equation*}

where $K$ is the number of perturbations (like image flipping) $\Psi ^{\eta }$ done. A value of $K=2$ is recommended by the authors. According to authors, the estimated pseudo-labels $\widehat {\boldsymbol {y}}{}_{j}$ might present high entropy, increasing low confidence estimations. To address this, the output array $\widehat {\boldsymbol {y}}$ is sharpened with a temperature coefficient $\rho $ (with $\rho =0.25$ recommended by the authors):

\begin{equation*} \widetilde {\boldsymbol {y}}_{i}=\frac {\widehat {y}_{i}^{1/\rho }}{\sum _{j}\widehat {y}_{j}^{1/\rho }}.\tag{7}\end{equation*}

The set $\widetilde {S}_{u}=\left ({X_{u},\widetilde {Y}}\right)$ corresponds to the data with the sharpened pseudo labels, where $\widetilde {Y}=\left \{{ \widetilde {\boldsymbol {y}}_{1},\widetilde {\boldsymbol {y}}_{2},\ldots,\widetilde {\boldsymbol {y}}_{n_{u}}}\right \}$

Authors in [31] highlight how data augmentation is important to improve the SSDL performance. Therefore the authors proposed the MixUp approach [32], which consists on augmenting data using both labelled and unlabelled observations: $\left ({S'_{l},\widetilde {S}'_{u}}\right)=\Psi _{\textrm {MixUp}}\left ({S_{l},\widetilde {S}_{u},\alpha }\right)$, where $S_{l}=\left ({X_{l},Y_{l}}\right)$ stands for the labelled data with a sample size of $n_{l}$. The MixUp algorithm generates new observations combining the unlabelled (with its pseudo labels) and labelled data through a linear interpolation. Specifically, for two labelled and/or pseudo labelled data pairs $\left ({\boldsymbol {x}_{a},y_{a}}\right)$ and $\left ({\boldsymbol {x}_{b},y_{b}}\right)$, the MixUp approach creates a new observation and its label $\left ({\boldsymbol {x}'=\lambda '\boldsymbol {x}_{a}+\left ({1-\lambda '}\right)\boldsymbol {x}_{b},y'=\lambda 'y_{a}+\left ({1-\lambda '}\right)y_{b}}\right)$ using a linear interpolation. The parameter $\alpha $ controls the Beta distribution where the MixUp coefficient is sampled from $\lambda \sim \textrm {Beta}\left ({\alpha,\alpha }\right)$. A value of $\alpha =0.75$ is recommended by the authors [31]. This results in the augmented data sets $\left ({S'_{l},\widetilde {S}'_{u}}\right)$, used by the MixMatch algorithm to train a model as specified in the training function $T_{\textrm {MixMatch}}$:

\begin{align*} f_{\boldsymbol {\theta }}=&T_{\textrm {MixMatch}}\left ({S_{l},X_{u},\gamma }\right)=\underset {\boldsymbol {w}}{\textrm {argmin}}~\mathcal {L}\left ({S,\boldsymbol {w}}\right) \tag{8}\\ \mathcal {L}\left ({S,\boldsymbol {\theta }}\right)=&\sum _{\left ({\boldsymbol {x}_{i},\boldsymbol {y}_{i}}\right)\in S'_{l}}\mathcal {L}_{l}\left ({\boldsymbol {\theta },\boldsymbol {x}_{i},\boldsymbol {y}_{i}}\right) \\&+\,\gamma r(\tau) \sum _{\left ({\boldsymbol {x}_{j},\widetilde {\boldsymbol {y}}_{j}}\right)\in \widetilde {S}'_{u}}\mathcal {L}_{u}\left ({\boldsymbol {\theta },\boldsymbol {x}_{j},\widetilde {\boldsymbol {y}}_{j}}\right) \tag{9}\end{align*}

In [31] the supervised loss term was implemented with a cross-entropy loss; $\mathcal {L}_{l}\left ({\boldsymbol {w},\boldsymbol {x}_{i},\boldsymbol {y}_{i}}\right)=\delta _{\textrm {cross-entropy}}\left ({\boldsymbol {y}_{i},f_{\boldsymbol {w}}\left ({\boldsymbol {x}_{i}}\right)}\right)$. Regarding the unlabelled loss term, an Euclidean distance was implemented $\mathcal {L}_{u}\left ({\boldsymbol {w},\boldsymbol {x}_{j},\widetilde {\boldsymbol {y}}_{j}}\right)=\left \Vert{ \widetilde {\boldsymbol {y}}_{j}-f_{\boldsymbol {w}}\left ({\boldsymbol {x}_{j}}\right)}\right \Vert $. Authors in [31], modelled the coefficient $r(\tau)$ as a ramp-up function that increases its value as the epochs $\tau $ increase. In our implementation, $r(\tau)$ was set to $\tau /3000$. The $\gamma $ factor is used as a regularization coefficient. It regulates the influence of unlabelled data. It is important to remark how unlabelled data also affects the *labelled* data term $\mathcal {L}_{l}$, as unlabelled data is used to augment data observations by using the MixUp approach for the labelled term as well.

### D. Semi and Self Supervised Learning for Improving Uncertainty Estimation

Recently, in [33] the authors analyze the use of unlabelled data to improve a model’s calibration (defined by the authors as the correlation between accuracy and uncertainty). A regularization based approach was implemented, improving the calibration of the model for structured data. Moreover, in [34], authors explore the improvement of uncertainty estimations using self-supervised learning. Some popular semi-supervised approaches like MixMatch [31] use concepts implemented in self-supervised learning, namely consistency regularization. The results presented in [33] reveal the advantage of using unlabelled data for uncertainty estimation. Semi-supervised learning has recently been proven to enhance adversarial robustness, as argued in [35]. Moreover, in [36], the impact of MixUp data augmentation on the model uncertainty estimation (also known as model calibration) is assessed. Authors used the Softmax function to estimate the model’s uncertainty, yielding better calibrations through the usage of MixUp. MixUp is also used in the MixMatch model [31].

### E. Comparing Model Uncertainty Reliability

To compare uncertainty reliability across different uncertainty estimation techniques, different approaches have been developed in the literature. Uncertainty reliability is related to the calibration error [37]. For a classification problem in a given data set $D$, intuitively, the calibration error refers to the difference between the total estimated probability (confidence) $\hat {p}$ for the observations of label $y$ and the real proportion of the estimation of a label $y$, given in $p$.

Reliability histograms [38] are proposed to build a histogram, with bins defined for different uncertainty ranges. A reliability histogram plots the normalized confidence against the accuracy for each bin. Defining $B_{m}$ as the set of indices of observations whose uncertainty prediction belongs to the interval $I_{m}=\left ({\frac {m-1}{M},\frac {m}{M}}\right]$, the sample mean accuracy for the bin $B_{m}$ is given by:

\begin{equation*} \overline {\textrm {acc}}\left ({B_{m}}\right)=\frac {1}{\left |{B_{m}}\right |}\sum _{i\in B_{m}}\boldsymbol {1}\left ({\hat {y}_{i}=y_{i}}\right),\tag{10}\end{equation*}

where $\hat {y}_{i}$ corresponds to the model estimation for the observation $i$ with label $y_{i}$. Similarly the average uncertainty for a bin $B_{m}$ for an uncertainty density function $\hat {p}$ is given by:

\begin{equation*} \overline {\textrm {unc}}\left ({B_{m}}\right)=\frac {1}{\left |{B_{m}}\right |}\sum _{i\in B_{m}}\hat {p}_{i}.\tag{11}\end{equation*}

An uncertainty estimator is considered better as the relationship of $\overline {\textrm {unc}}$ and $\overline {\textrm {acc}}$ reaches the identity and thus becomes less spiky. The Expected Calibration Error (ECE) measures this gap in one scalar, taking the average difference between the sample accuracy and confidence mean:

\begin{equation*} \text {ECE}=\sum _{m=1}^{M}\frac {\lvert B_{m}\rvert }{n}\lvert \overline {\text {acc}}(B_{m})-\overline {\text {conf}}(B_{m})\rvert.\tag{12}\end{equation*}

In [37] different downsides of the ECE are noted. One such downside is the sparseness that is frequently yielded by the computed confidence histogram. This is referred in [37] as the problem of fixed calibration ranges. Frequently used Softmax based uncertainty estimations are overconfident, making higher bins more populated. This makes the estimates of less populated bins potentially inaccurate. Other improvements added to the ECE include the root mean squared calibration error [39] and the static and adaptive calibration error [37].

However, an important downside of using the ECE is the assumption that it makes about the uncertainty measurement as a normalized measure between 0 and 1. Different approaches for uncertainty estimation as MCD and DUQ yield unbounded values (outside from the 0 to 1 interval), making the use of the ECE inappropriate. For instance in [26], MCD has been implemented for uncertainty estimation, with no normalized values reported. For instance, comparing uncertainty estimations of [25], [26] to the ones yielded in [40], is difficult as different uncertainty measures yield different uncertainty value ranges for different data sets. Using the ECE is only possible when a bounded uncertainty estimator such as the Softmax function is used (where its values are bounded from 0 to 1). This makes the comparison of uncertainty estimation approaches difficult as they can be normalized using the sampled values for the data set tested, but this leads to a data set bias.

However, a bigger issue when using a measure like the ECE is the limited statistical interpretation. The ECE relies on the sample mean per bin $\overline {\text {acc}}$, which ignores the distribution of the data and information from other statistical measurements like the variance.

As an alternative, rejection-classification plots were used in [22]. Rejection-classification plots use as x-axis the proportion of data rejected based on the uncertainty score. The y-axis represents the level of accuracy. Similar to the rejection-classification plots, the accuracy vs. confidence curves were used in [23] to compare different uncertainty estimators graphically. For a quantitative comparison, the area under the curve of this plot can be used. However, such value is also unbounded and holds no statistical significance. For either the rejection plots or the ECE based metrics, a comparison problem arises when the compared curves present different accuracy levels. As the number of wrong estimations fluctuates for each model, the average accuracy per bin also changes, making it harder to compare the uncertainty estimation quality. This situation is faced in this work, where we compare the impact of a supervised to a semi-supervised model, which changes the model’s accuracy.

Other common metrics to measure the error of a model have also been used for out of distribution data detection through uncertainty methods. In [41] for instance, the area under the precision-recall curve and the error rate have both been used for out of distribution detection to compare uncertainty estimation methods. However, the metric is also not statistically relevant as no distribution information is used to compare the evaluated methods. Using the outlined context, this work comprises the following contributions:

• We explore the impact of semi-supervised deep learning in the reliability of the uncertainty estimations for COVID-19 detection, using a common deep learning architecture.
• We evaluate and compare qualitatively as well as quantitatively the performance of three different uncertainty estimation techniques for both the supervised and semi-supervised models.
• We propose the use of the Jensen-Shannon divergence [42] as a probability density based metric to compare the performance of uncertainty estimation techniques.

We show that our proposed method is simple to implement and that it is often effective. The method takes advantage of unlabelled data to improve uncertainty estimations for COVID-19 detection using digital chest X-ray images. Unlabelled data is generally widely available, and in the context of a virus out-break, easier to obtain, when compared to labelled data.

## III. Proposed Method

In this work, we propose the use of unlabelled data through MixMatch (as depicted in equations 8 and 9), to improve uncertainty estimation. We test the impact of using unlabelled data in three uncertainty estimation methods:

• Softmax as described in Equation 1, using the maximum Softmax value for the output layer. Therefore, the Softmax uncertainty estimation corresponds to $u_{i}=\mathop{\arg \max }\limits_{i}\: p_{i}$.
• MCD as depicted in Equation 3, using the standard deviation of the distribution from the evaluation of the model with dropout for the same input observation $\boldsymbol {x}_{j}$ [19], making $u_{i}=\sigma _{\textrm {model}}\left ({\boldsymbol {x}_{j}}\right)$.
• DUQ as introduced in Equation 4. We used a generic weight matrix $W_{i} = 1$ for all classes $i=1,\ldots,C$, implementing an Euclidean distance for the radial basis kernel $K_{i}$. The uncertainty estimation for this approach is implemented as
\begin{equation*} u_{i} = \underset {i}{\arg \max }\: K_{i}\left ({f_{\boldsymbol {\theta }}\left ({\boldsymbol {x}_{j}}\right),\boldsymbol {e}_{i}}\right),\tag{13}\end{equation*}
  for an input observation $\boldsymbol {x}_{j}$.

**FIGURE 1.**
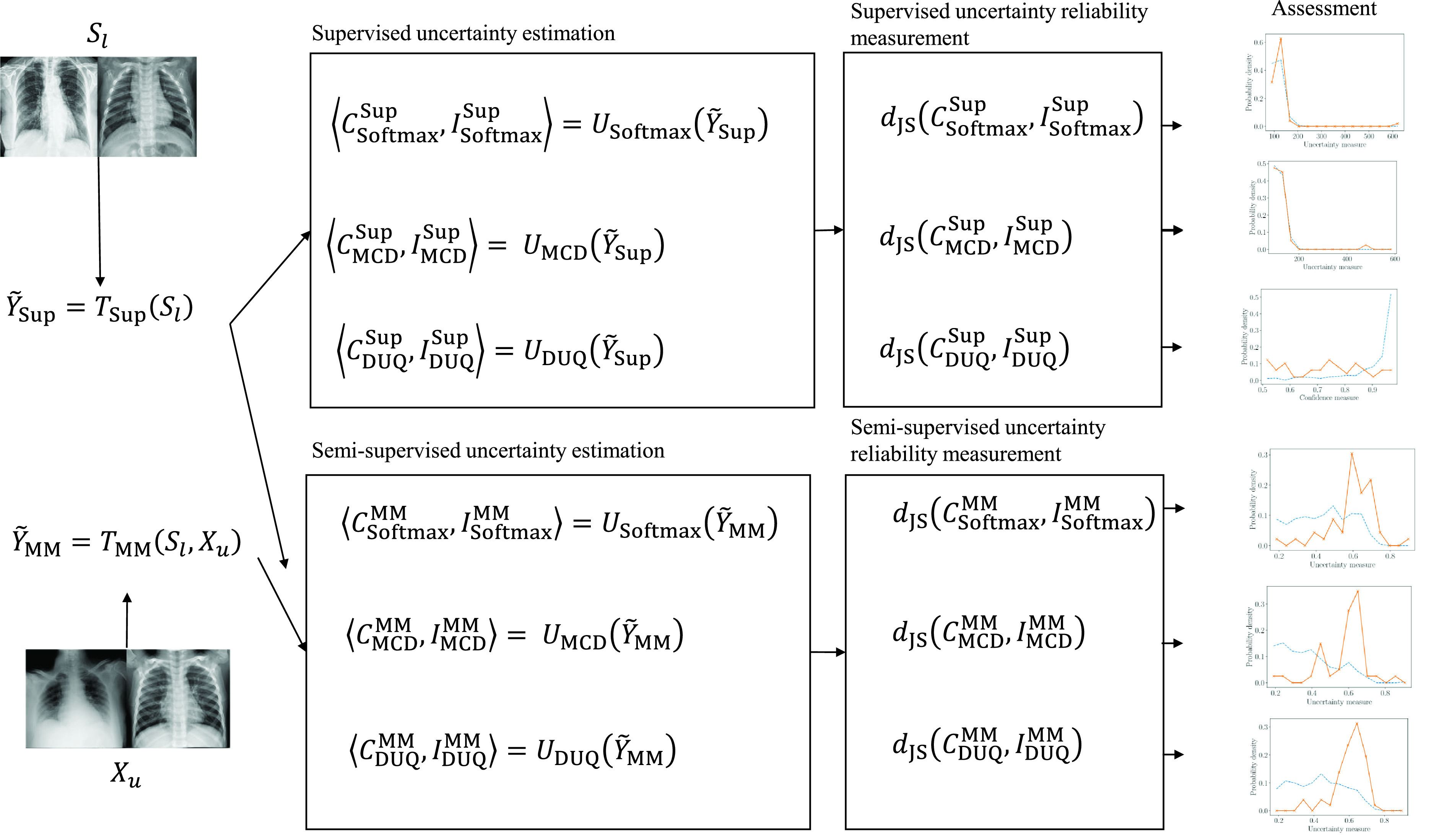
Description of the implemented work-flow: Training of the semi-supervised model MixMatch (MM) and the supervised model (Sup.). Calculation of the predictive uncertainties using the Softmax activation function, MonteCarlo Dropout (MCD) and Deterministic Uncertainty Quantification (DUQ). We propose to compare the distribution of the predictive uncertainties for correct (C) and Incorrect (I) estimations, using the Jensen-Shannon distance (JS).

In this work we also propose the comparison of the evaluated methods for uncertainty estimation, using the Jensen-Shannon divergence between the distribution of the uncertainty estimations for the correct and incorrect estimations. More specifically, take a model uncertainty estimation $u_{j}$ for an input observation $\boldsymbol {x}_{j}$. For a given data set $S$, we group the uncertainties of the wrong estimations for the trained model, semi or supervised, as wrong or correct according to the labels in the test partition of the labelled dataset $S_{l}=\left ({X_{l},Y_{l}}\right)$. This results in a set of uncertainties for the wrong estimations $U_{\textrm {wrong}}=\left \{{ u_{1},\ldots,u_{n_{\textrm {wrong}}}}\right \} $ and correct estimations $U_{\textrm {correct}}=\left \{{ u_{1},\ldots,u_{n_{\textrm {incorrect}}}}\right \} $, used to calculate the corresponding normalized histograms $\boldsymbol {p}_{u_{\textrm {correct}}}$ and $\boldsymbol {p}_{u_{\textrm {incorrect}}}$. We implement the Jensen-Shannon divergence $D_{\textrm {JS}}\left ({\boldsymbol {p}_{u_{\textrm {correct}}},\boldsymbol {p}_{u_{\textrm {incorrect}}}}\right)$ to measure the divergence between the two non-parametric approximations of the density functions $\boldsymbol {p}_{u_{\textrm {correct}}}$ and $\boldsymbol {p}_{u_{\textrm {incorrect}}}$. Figure 1 summarizes the implemented pipeline in this work.

## IV. Experiments

### A. Dataset

The COVID-19^+^ data sample was downloaded from the publicly available github repository of Cohen [28]. The observations were gathered from journals such as radiopaedia.org and the Italian Society of Medical and Interventional Radiology. In this work we used only images labelled with COVID-19^+^, discarding images labelled as Middle East Respiratory Syndrome (MERS), Acute Respiratory Distress Syndrome (ARDS) and Severe Acute Respiratory Syndrome (SARS). After applying this filtering, 99 observations of front chest X-rays were selected. The images were stored with resolutions ranging from $400 \times 400$ up to $2500 \times 2500$ pixels.

Together with the COVID-19^−^ observations we sampled a 5856 observations containing pneumonia and no lung pathology’s as defined by [29]. The data set is composed of 4273 observations of viral and bacterial pneumonia and 1583 normal observations (with no lung pathology). We used the observations with no findings, for the COVID-19^−^ class. The negative COVID-19 cases gathered in this dataset have been used in recent research related to COVID-19 detection using deep learning [43]–[45]. The images were stored with a resolution of $1300 \times 600$ pixels.

**FIGURE 2.**
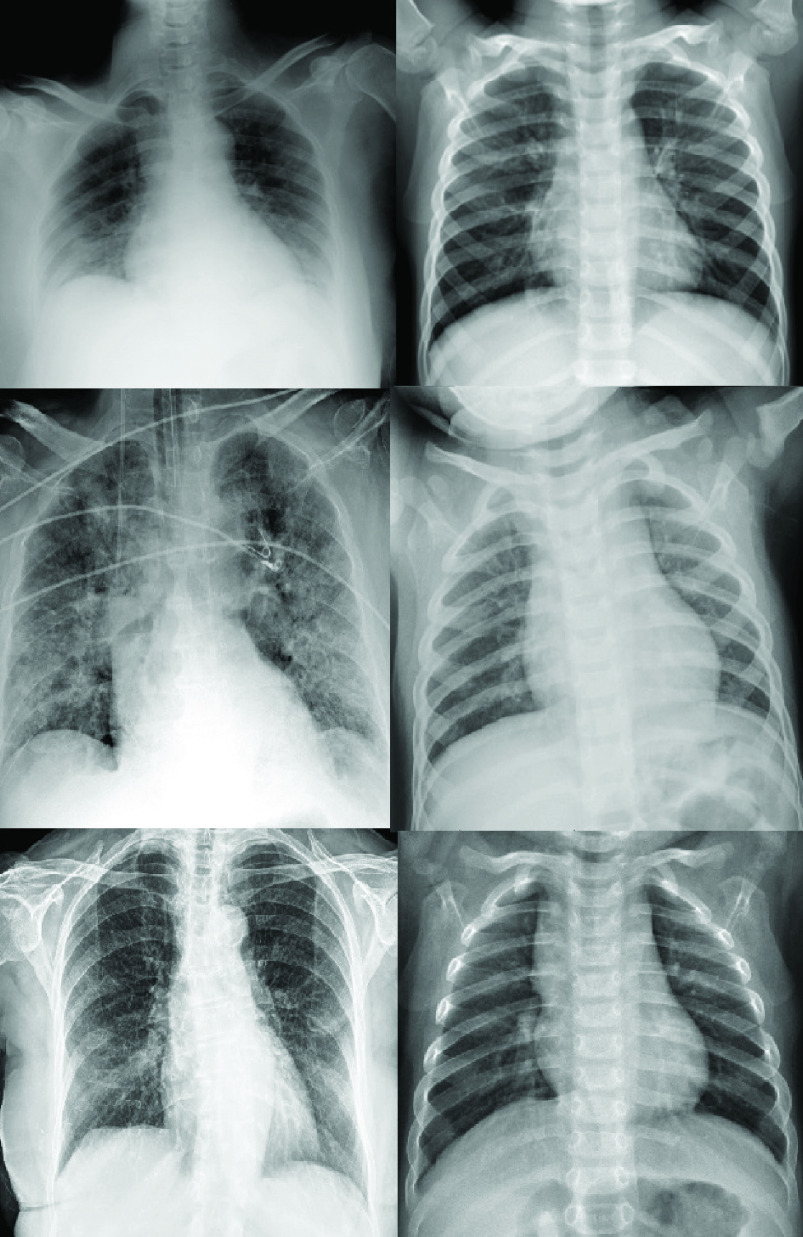
Left column, positive COVID-19 X-ray observations, right column, three negative COVID-19 observations. All of them were taken from the dataset used in this work.

We created a balanced base-line data set of 99 COVID-19^+^ observations and also 99 observations for COVID-19^−^ cases, using the aforementioned data sets. Figure 2 shows a sample of the images used.

Both supervised and semi-supervised models were trained with $n_{l}=20, 30, 60, 70, 100$ labelled observations, to study the impact of different labelled data sample sizes. We splitted the data set of 198 observations with 70% (138 observations) of the data for training and the remaining 30% (60 observations) for testing. The labelled observations were taken from the training dataset, and as for the SSDL model, we used the remaining as unlabelled data, always keeping the number of labels balanced. We chose to use the unlabelled data as a partition of the original labelled dataset, to avoid distribution mismatch related issues as suggested in [46]. This is out of the scope in this work, however testing unlabelled datasets from other sources with possibly more observations, is left for future work.

### B. Neural Network Architectures and Metrics

In this work we used a WideResNet model as a supervised model for binary classification (COVID-19^+^ and COVID-19^−^ discrimination), with transfer learning from the ImageNet dataset. For the supervised model we used the cross entropy as loss function. The semi-supervised MixMatch framework implemented also used the WideResNet model with a $K=2$ transformations, a sharpening coefficient $\rho =0.25$, a MixUp parameter $\alpha = 0.75$, as recommended in [31], and a $\gamma = 200$ for the unsupervised coefficient, as advised in [11]. For both the supervised and semi-supervised model we used a learning rate of 0.00002 and a batch size of 10 observations, with 50 epochs per run. As a preprocessing stage, we implemented a standardization of the training dataset. All images were resized to $150\times 150$ pixels. The model was implemented with the FastAI library, and optimized with the 1-cycle policy [47].

We evaluated the Softmax, MCD and DUQ uncertainty methods in the semi and supervised models to collect for each one of them a set of uncertainties $U_{\textrm {S}}$, $U_{\textrm {MCD}}$, and $U_{\textrm {DUQ}}$, respectively. As for the parameters of the tested uncertainty methods, for the MCD, we used $M=100$ evaluations with the default dropout of WideResNet. Regarding the DUQ method, we used an Euclidian based kernel $K$ for all the classes.

We first report the model’s F1 score, to compare the accuracy gained when using SSDL, and use it as a reference for the uncertainty results analysis. This is depicted in Table 1. We also report the $\rho _{\textrm {lu}}$ and $\delta _{\rho }$ (this last one for the SSDL model), as advised in [11] for assessing the accuracy gain for SSDL frameworks.

**TABLE 1:** F1 Score and Accuracy Statistics for Batches Tested With Different Number of Labels $n_{l}$

$n_{l}$	Desc. stat.	No SSDL F1-Score/Accuracy	SSDL F1-Score/Acc	$\rho _{lu}$	$\Delta _{\rho }$
20	$\overline {x}$	0.754/0.816	0.93/0.965	0.144	7.684
	s	0.069/0.07	0.0415/0.021		
30	$\overline {x}$	0.836/0.89	0.943/0.976	0.217	7.295
	s	0.07/0.048	0.032/0.013		
60	$\overline {x}$	0.902/0.96	0.917/0.971	0.434	0.641
	s	0.044/0.028	0.048/0.018		
70	$\overline {x}$	0.931/0.958	0.909/0.968	0.507	0.409
	s	0.032/0.025	0.0473/0.025		
100	$\overline {x}$	0.932/0.975	0.791/0.9	0.724	−2.287
	s	0.032/0.018	0.064/0.029		

Secondly, we report the sample mean and standard deviation for the correct and incorrect estimations. We perform this comparison for all three tested uncertainty estimation methods (Softmax, MCD and DUQ). We also measure the Jensen-Shannon divergence between the distributions of the uncertainties $\boldsymbol {p}_{u_{\textrm {incorrect}}}$ and $\boldsymbol {p}_{u_{\textrm {correct}}}$, for the incorrect and correct estimations, respectively. Uncertainty for wrong and right estimations is expected to be higher and lower respectively. The reported descriptive statistics correspond to the results of 10 runs with 10 different test and training data partitions. The results yielded for the described experiment are displayed in tables 2, 3 and 7.

**TABLE 2:** Softmax Results for the Semi-Supervised and Supervised Models With Different Numbers of Labels $n_{l}$. Higher Values Indicate Higher Model Confidence. The Higher the Better for Correct Estimations, and the Lower the Better for Incorrect Estimations

$n_{l}$	Desc. stat.	$s({y})$ correct NO-SSDL	$s({y})$ wrong NO-SSDL	JS div. No SSDL	$s({y})$correct SSDL	$s({y})$ wrong SSDL	JS div. No SSDL
20	$\overline {x}$	0.8216	0.7343	0.2407	0.9452	0.7481	0.5812
	s	0.1384	0.1457		0.0932	0.1298	
30	$\overline {x}$	0.8594	0.7207	0.3554	0.9597	0.7722	0.5497
	s	0.1337	0.1429		0.0755	0.1556	
60	$\overline {x}$	0.9159	0.7128	0.506587	0.9301	0.7315	0.5462
	s	0.1116	0.1504		0.0986	0.1384	
70	$\overline {x}$	0.9097	0.7387	0.4514	0.9232	0.7309	0.4976
	s	0.1167	0.1535		0.1066	0.1467	
100	$\overline {x}$	0.9324	0.7297	0.5067	0.8445	0.7254	0.2872
	s	0.1016	0.1471		0.1438	0.1505

**TABLE 3:** MCD Results for the Semi-Supervised and Supervised Models With Different Numbers of Labels $n_{l}$. Lower Values Indicate Higher Model Confidence. The Lower the Better for Correct Estimations, and the Higher the Better for Incorrect Estimations

$n_{l}$	Desc. stat.	$\sigma $ correct No SSDL	$\sigma $ wrong No SSDL	JS div. No SSDL	$\sigma $ correct SSDL	$\sigma $ wrong SSDL	JS div. SSDL
20	$\overline {x}$	0.5401	0.6222	0.271	0.3076	0.6334	0.4938
	s	0.1266	0.0852		0.2107	0.0925	
30	$\overline {x}$	0.5008	0.6370	0.367503	0.2890	0.6038	0.5204
	s	0.1552	0.0964		0.1904	0.1255	
60	$\overline {x}$	0.4041	0.6253	0.49075	0.3687	0.6317	0.5591
	s	0.1856	0.1150		0.1975	0.0730	
70	$\overline {x}$	0.4133	0.6253	0.4301	0.3968	0.6355	0.5105
	s	0.1882	0.1228		0.1825	0.0840	
100	$\overline {x}$	0.3540	0.6186	0.5534	0.5179	0.6313	0.3382
	s	0.2028	0.1348		0.1551	0.0903

**TABLE 4:** DUQ Uncertainty Distributions for Correct (Blue Dashed Line) and Incorrect Estimations (Orange Dashed With ‘x’ Line) Using $n_{l} = 30, 70$ Labels (From Left to Right). From Top to Bottom, the First Row Corresponds to the Supervised Model and the Second Row, to the SSDL Model Results

**TABLE 5:** MCD Uncertainty Distributions for Correct (Blue Dashed Line) and Incorrect (Orange Dashed With ‘x’ line) Estimations Using $n_{l} = 30, 70$, From Left to Right. From Top to Bottom, the Supervised and the Semi-Supervised Deep Learning Models Results

**TABLE 6:** Softmax Confidence Distributions for Correct (Blue Dashed Line) and Incorrect (Orange Dashed With ‘x’ Line) Estimations Using $n_{l} = 30, 70$, From Left to Right. From Top to Bottom, the Supervised and the Semi-Supervised Deep Learning Models Results

**TABLE 7:** DUQ Results for the Semi-Supervised and Supervised Models With Different Numbers of Labels $n_{l}$. Lower Values Indicate Higher Model Confidence. The Lower the Better for Correct Estimations, and the Higher the Better for Incorrect Estimations

$n_{l}$	Desc. stat.	$\ell _{2}$ correct No SSDL	$\ell _{2}$ wrong No SSDL	JS div.No SSDL	$\ell _{2}$ correct SSDL	$\ell _{2}$ wrong SSDL	JS div. SSDL
20	$\overline {x}$	133.7481	143.8356	0.072	131.9169	137.8194	0.17024
	s	24.4915	55.7149		33.7233	21.8417	
30	$\overline {x}$	130.7032	147.3737	0.13599	132.2328	142.4301	0.108488
	s	32.4645	48.0116		31.6105	58.383	
60	$\overline {x}$	135.0814	137.0349	0.072	132.7359	142.3879	0.163092
	s	35.8013	20.6742		37.7423	20.4047	
70	$\overline {x}$	132.1344	149.7608	0.216469	132.5677	148.2541	0.14228
	s	24.8133	80.2325		32.204	75.853	
100	$\overline {x}$	134.1262	143.4125	0.1599	132.6018	140.198	0.0943
	s	29.4393	71.2851		34.5869	51.501	

Finally, as a complementary qualitative test, we calculated the rejection-classification plots described in [22]. The average accuracy was calculated for each uncertainty bin. In general, for rejection plots, the less spiky and closer to an identity function, the better for an uncertainty estimator. Such plots are displayed in Table 9 for the three tested uncertainty estimators.

**TABLE 8:** Summary of the Jensen-Shannon Divergence Gains (Uncertainty Distributions Divergence for the Correct and Incorrect Estimations), for Each Tested Uncertainty Estimation Method, Using Semi-Supervised Learning

$n_{l}$	MCD: JS div. gain SSDL vs. No SSDL	Softmax: JS div. gain SSDL vs. No SSDL	DUQ: JS div. gain SSDL vs. No SSDL
20	+0.222/+81%	+0.34/+142%	+0.098/+136%
30	+0.153/+41.7%	+0.194/+54.6%	−0.027/−20%
60	+0.069/+14%	+0.04/+8%	+0.092/+126%
70	+0.08/+18%	+0.04/+10%	−0.073/−34%
100	−0.21/−38%	−0.22/−0.43%	−0.065/−41%

**TABLE 9:** Rejection Plots for the Three Tested Uncertainty Approaches. The First Row Correspond to the DUQ Estimations, the Second One to the MCD Uncertainties and the Last One to the Softmax Confidence Scores. From Left to Right, Models With Different Number of Labels $n_{l}$. Orange and ‘x’ Lines Correspond to the Semi-Supervised Model and the Dashed and Blue Lines Correspond to the Supervised Model

$n_{l}=20$	$n_{l}=60$	$n_{l}=70$
		
		
		

## V. Results

The F1-score and accuracy of the models trained with less than 70 labels reported a significant performance gain when using the tested SSDL model. The F1-score gain goes from around 0.18 with 20 labels to almost 0.01 when using 60 labels. With 70 labels, the sample mean accuracy and F1-score gets marginally better for the supervised model, making the impact of SSDL negligible. We also report the $\Delta _{\rho }$ to measure the accuracy gain under the specific SSDL data setting. The yielded results allow to evaluate uncertainty estimation performance under the setting of substantial ( $n_{l}=20,30$), marginal ( $n_{l}=60$) and negative ( $n_{l}=70, 100$) accuracy and F1-score gains when using MixMatch.

Taking the accuracy gains into account for different number of labels $n_{l}$ used for training we proceed to analyze the uncertainty estimation reliability by using the proposed Jensen-Shannon divergence between the uncertainty distribution of correct and wrong estimations.

For the Softmax function, the wrong-correct uncertainty distribution distances are depicted in Table 2. In this table, a significant Jensen-Shannon divergence gain is yielded when $n_{l}=20$ and $n_{l}=30$, with gains ranging from 0.32 to 0.2. However, when $n_{l}=60$ and $n_{l}=70$ the Jensen-Shannon (JS) divergence gets smaller between the supervised and SSDL model, with only 0.04 of difference. For $n_{l}=100$, the supervised model gets a much higher JS divergence, suggesting a high correlation between the accuracy/f1-score gain and uncertainty reliability gain by using SSDL for the softmax uncertainty based approach. Table 6 shows how the distributions of the softmax uncertainties for the wrong and correct distributions are significantly different for both the SSDL and supervised models, however, the JS divergence makes easier to spot the difference between the distributions quantitatively.

As for the MCD for uncertainty estimation, a similar behavior can be observed, with decreasing uncertainty reliability gains when the number of labels go from $n_{l} = 20$ and $n_{l} = 100$ when using the SSDL model. Similarly, for $n_{l} = 20$ up to $n_{l} = 70$ labels, the reliability of the SSDL model uncertainty estimations outperform the supervised model by a larger margin. MCD obtains lower reliability gains for the SSDL model when compared to the Softmax approach, for the lowest number of labels $n_{l} = 20$ and $n_{l} = 30$ tested. Also for the SSDL model, when the number of labels increases from $n_{l}= 60$, the reliability of the MCD approach is better when compared to the softmax method. The uncertainty distribution plots for the correct and wrong estimations depicted in Table 5 show important differences between such distributions, but the improvement between the SSDL and supervised models is hard to discern visually.

Regarding the results for the DUQ uncertainty estimation method, the overall JS divergences are significantly lower than the MCD and softmax approaches. This suggests that both methods significantly outperform DUQ as an uncertainty estimation method. The plots in Table 4 qualitatively complement the small difference between the DUQ uncertainty distributions of correct and wrong estimations. However, similar to the softmax and MCD methods, the usage of SSDL makes a positive impact when $n_{l}$ is between $n_{l} = 20$ up to $n_{l} = 60$.

A summary of the results is presented in Table 8. The use of the Jensen-Shannon divergence between the uncertainty distributions of the correct and wrong estimations allowed us to perform such analysis. We can see how the highest relative uncertainty estimation improvements are yielded when the models are trained with fewer labels. In such case, the gains range from 81 to 142 percent, for all the tested uncertainty estimation methods. In general, as the number of labels increases, the reliability gain of the uncertainty estimations using SSDL tend to decrease. This correlates well with the average accuracy gains using SSDL depicted in Table 1.

This tendency is more visible for the MCD and Softmax methods. The DUQ method is very unstable, as its capability for uncertainty estimation is more limited when compared to the first two methods, with lower JS divergences for all the tested configurations as seen in Table 7. Marginal uncertainty estimation improvements were obtained for the DUQ method, as seen in Table 8.

Finally, Table 9 shows the rejection plots for the tested uncertainty estimation methods, with different numbers of labels $n_{l}$. In most cases the plots are rather similar, and also reveal a very high dispersion of the results for each bin, depicted by the blue (supervised model) and the orange areas (SSDL model). Such high dispersion suggests a possible statistically irrelevant comparison of results. Most of the plotted curves show higher accuracies per bin for the SSDL model, which corresponds to the results yielded in Table 1 where for most tested configurations the SSDL model outperforms the supervised one. This makes the comparison of the rejection plots between the supervised and the SSDL model harder.

## VI. Limitations of the Study

This work used a limited sample of COVID-19 positive observations coming from a very different distribution when compared to the source of COVID-19 negative observations sampled from [29]. This causes a bias in the population of patients sampled for COVID-19 positive and negative cases related to age and ethnicity, as the data sources for both cases are completely different. The low availability of public repositories of COVID-19 chest X-rays with reliable labels at the time of writing poses a limitation to this work. Therefore, an additional validation of the proposed method in this work with other datasets with higher quality (with less age and ethnicity biases) is necessary. We plan to do this in the future. This work focused on measuring the impact of semi-supervised learning on uncertainty estimations for COVID-19 detection, and evidenced how predictive uncertainty estimations improve as model accuracy improves. However, the quality of the predictive uncertainty estimations can be improved through model calibration methods. Furthermore, other uncertainty estimation methods can be included in the comparison. We plan to test uncertainty estimation improvements in future work.

## VII. Conclusion

In this work we have tested the impact of using unlabelled data to improve the reliability of uncertainty estimations through the implementation of the SSDL algorithm known as MixMatch. We tested three different uncertainty estimation methods (softmax, MCD and DUQ). The yielded descriptive statistics suggest an important reliability improvement of the uncertainty estimations when using SSDL for all the three uncertainty estimation methods. With low number of labels, the JS divergence is boosted by up to 142%, as seen in Table 8.

To ease the comparison of the tested uncertainty techniques, we proposed the use of the JS divergence, comparing the distributions of the wrong and correct estimations. The test is statistically relevant as it takes into account the whole results distribution, and it is easy to interpret, with values ranging from 0 to 1 (the higher the values the better). The use of the JS divergence index to compare the uncertainty estimations proved to be simple to analyze, with easy to map correspondence with the distribution plots. Its use is recommended when comparing different uncertainty methods under different models which cause fluctuations in the model accuracy.

When comparing the three tested uncertainty estimation methods, the MCD and the softmax techniques performed better than the DUQ approach. The comparison between the MCD and the softmax methods is rather mixed, with MCD performing better when $n_{l}$ is higher. Results with the DUQ method yielded a significantly worse performance for uncertainty estimation. We speculate that this is due to the high similarity between the images of the two classes. This makes the averaged observations in the feature space similar for both classes and the comparison of new unseen observations less sensitive. In terms of the uncertainty source, the MCD approach seemed to be more sensitive to epistemic uncertainty, than the DUQ method. Epistemic uncertainty can be considered to be very high in models trained with very few labels, as the feature space sample is very limited. MCD takes into account the epistemic uncertainty of both the feature extractor and the top model (fully connected network acting as classifier), unlike DUQ which only uses the feature extractor, and can be considered the only channel for the epistemic uncertainty for this method.

As future work, we plan to explore more recent uncertainty estimation approaches which have been originally designed for distribution mismatch measurement [48], [49]. Interchangeably, the quality of the unlabelled dataset and its impact in the model’s accuracy and uncertainty estimations is also worth to explore. For this end, dataset quality metrics can be implemented [50]. Furthermore, we plan to explore the impact of unlabelled data in other engineering requirements of deep learning models such as model robustness. Little research has been done about the actual impact of semi or self supervised learning in important model properties such as robustness in practical applications like medical imaging analysis. For instance, we plan to further evaluate the improvement of model uncertainty reliability and robustness for COVID-19 detection using computed tomography as an alternative imaging technology which is also interesting to explore. The use of modern semi- and self-supervised techniques can do more than just improving the model accuracy under restricted number of labels. Therefore its impact should be studied in depth. In general, we highlight the need for evaluating other important model properties such as robustness and uncertainty reliability, specially for sensitive applications like medical imaging analysis.
